# Bis{μ-1,3-bis­[(benzimidazol-1-yl)meth­yl]benzene-κ^2^ *N* ^3^:*N* ^3′^}bis­[dichloridozinc(II)] dimethyl­formamide disolvate

**DOI:** 10.1107/S1600536809015943

**Published:** 2009-05-07

**Authors:** Li-Zhen Zhao, Ping Li, Bao-Liang Cao, Seik Weng Ng

**Affiliations:** a Department of Chemistry, Jining Normal College, Wulanchabu, Inner Mongolia 012000, People’s Republic of China; b Department of Chemistry, University of Malaya, 50603 Kuala Lumpur, Malaysia

## Abstract

In the title compound, [Zn_2_Cl_4_(C_22_H_18_N_4_)_2_]·2C_3_H_7_NO, the 1,3-bis­[(benzimidazol-1-yl)meth­yl]benzene ligand bridges two ZnCl_2_ units, forming a centrosymmetric dinuclear mol­ecule. The Zn^II^ atom shows a distorted tetra­hedral coordination within a Cl_2_N_2_ donor set.

## Related literature

For the crystal structure of 1,3-bis­((benzimidazol-1-yl)meth­yl)benzene, which was isolated as the malonic acid co-crystal, see: Aakeröy *et al.* (2005 ▶). For related metal complexes, see: Fan *et al.* (2006 ▶); Raehm *et al.* (2003 ▶).
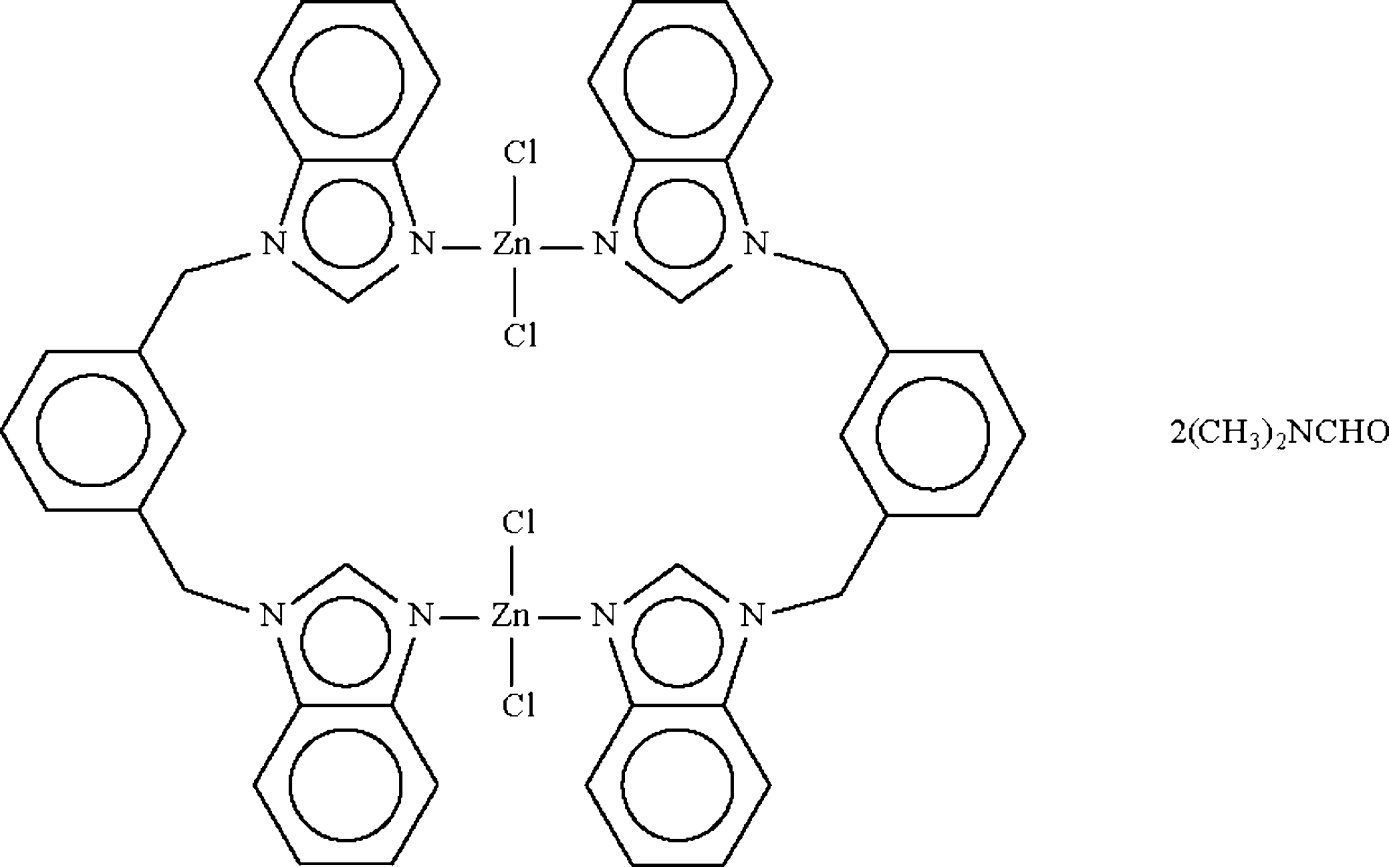


## Experimental

### 

#### Crystal data

[Zn_2_Cl_4_(C_22_H_18_N_4_)_2_]·2C_3_H_7_NO
*M* *_r_* = 1095.54
Monoclinic, 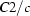

*a* = 24.0069 (5) Å
*b* = 9.8217 (2) Å
*c* = 23.9723 (5) Å
β = 117.695 (1)°
*V* = 5004.8 (2) Å^3^
*Z* = 4
Mo *K*α radiation
μ = 1.22 mm^−1^
*T* = 120 K
0.30 × 0.20 × 0.10 mm

#### Data collection

Bruker SMART APEX diffractometer
Absorption correction: multi-scan *SADABS* (Sheldrick, 1996 ▶) *T*_min_ = 0.711, *T*_max_ = 0.888
17166 measured reflections
5730 independent reflections
4449 reflections with *I* > 2σ(*I*)
*R*_int_ = 0.034

#### Refinement

*R*[*F* ^2^ > 2σ(*F* ^2^)] = 0.037
*wR*(*F* ^2^) = 0.097
*S* = 1.04
5730 reflections
309 parameters
H-atom parameters constrained
Δρ_max_ = 0.92 e Å^−3^
Δρ_min_ = −0.57 e Å^−3^

### 

Data collection: *APEX2* (Bruker, 2008 ▶); cell refinement: *SAINT* (Bruker, 2008 ▶); data reduction: *SAINT*; program(s) used to solve structure: *SHELXS97* (Sheldrick, 2008 ▶); program(s) used to refine structure: *SHELXL97* (Sheldrick, 2008 ▶); molecular graphics: *X-SEED* (Barbour, 2001 ▶); software used to prepare material for publication: *publCIF* (Westrip, 2009 ▶).

## Supplementary Material

Crystal structure: contains datablocks global, I. DOI: 10.1107/S1600536809015943/tk2441sup1.cif

Structure factors: contains datablocks I. DOI: 10.1107/S1600536809015943/tk2441Isup2.hkl

Additional supplementary materials:  crystallographic information; 3D view; checkCIF report

## Figures and Tables

**Fig. 1.**
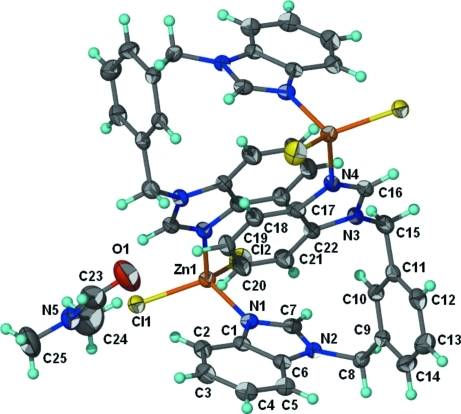
Thermal ellipsoid plot (Barbour, 2001) of [ZnCl2(C22H18N4)]2.2DMF at the 70% probability level; hydrogen atoms are drawn as spheres of arbitrary radius. Only one DMF molecule is shown. Unlabelled non-H atoms are related by a centre of inversion.

## supplementary crystallographic information

### Experimental

The compound was prepared from a mixture of boric acid (0.17 g), zinc chloride (0.27 g), 1,3-bis((benzimidazol-1-yl)methyl)benzene (0.45 g) in DMF (3.6 ml) and water (0.2 ml). The mixture was sealed in 25-ml Teflon-lined stainless-steel vessel, which was heated at 423 K for 5 days. The vessel was then cooled to room temperature slowly. Crystals were picked out manually.

### Refinement

Carbon-bound H-atoms were placed in calculated positions (C—H 0.95 to 0.99 Å) and were included in the refinement in the riding model approximation, with *U*(H) fixed at 1.2*U*_eq_(C).

### Crystal data

**Table:**

[Zn_2_Cl_4_(C_22_H_18_N_4_)_2_]·2C_3_H_7_NO	*F*(000) = 2256
*M**_r_* = 1095.54	*D*_x_ = 1.454 Mg m^−^^3^
Monoclinic, *C*2/*c*	Mo *K*α radiation, λ = 0.71073 Å
Hall symbol: -C 2yc	Cell parameters from 4135 reflections
*a* = 24.0069 (5) Å	θ = 2.3–27.2°
*b* = 9.8217 (2) Å	µ = 1.22 mm^−^^1^
*c* = 23.9723 (5) Å	*T* = 120 K
β = 117.695 (1)°	Prism, colorless
*V* = 5004.8 (2) Å^3^	0.30 × 0.20 × 0.10 mm
*Z* = 4	

### Data collection

**Table:**

Bruker SMART APEX diffractometer	5730 independent reflections
Radiation source: fine-focus sealed tube	4449 reflections with *I* > 2σ(*I*)
graphite	*R*_int_ = 0.034
ω scans	θ_max_ = 27.5°, θ_min_ = 1.9°
Absorption correction: multi-scan *SADABS* (Sheldrick, 1996)	*h* = −30→31
*T*_min_ = 0.711, *T*_max_ = 0.888	*k* = −12→12
17166 measured reflections	*l* = −31→28

### Refinement

**Table:**

Refinement on *F*^2^	Primary atom site location: structure-invariant direct methods
Least-squares matrix: full	Secondary atom site location: difference Fourier map
*R*[*F*^2^ > 2σ(*F*^2^)] = 0.037	Hydrogen site location: inferred from neighbouring sites
*wR*(*F*^2^) = 0.097	H-atom parameters constrained
*S* = 1.04	*w* = 1/[σ^2^(*F*_o_^2^) + (0.0452*P*)^2^ + 5.1375*P*] where *P* = (*F*_o_^2^ + 2*F*_c_^2^)/3
5730 reflections	(Δ/σ)_max_ = 0.001
309 parameters	Δρ_max_ = 0.92 e Å^−^^3^
0 restraints	Δρ_min_ = −0.57 e Å^−^^3^

### Fractional atomic coordinates and isotropic or equivalent isotropic displacement parameters (Å^2^)

**Table:**

	*x*	*y*	*z*	*U*_iso_*/*U*_eq_	
Zn1	0.499493 (12)	0.24704 (3)	0.615569 (12)	0.01976 (9)	
Cl1	0.54858 (3)	0.40106 (6)	0.69289 (3)	0.02422 (14)	
Cl2	0.54751 (3)	0.05513 (6)	0.61286 (3)	0.03540 (17)	
O1	0.28873 (14)	0.6931 (3)	0.61655 (11)	0.0738 (8)	
N1	0.46559 (9)	0.32960 (19)	0.52860 (9)	0.0194 (4)	
N2	0.42352 (9)	0.33960 (19)	0.42428 (9)	0.0183 (4)	
N3	0.16359 (9)	0.2139 (2)	0.36682 (9)	0.0223 (4)	
N4	0.08242 (9)	0.2789 (2)	0.38075 (9)	0.0224 (4)	
N5	0.37081 (12)	0.8246 (2)	0.67983 (10)	0.0369 (6)	
C1	0.43461 (10)	0.4540 (2)	0.50863 (10)	0.0185 (5)	
C2	0.42823 (11)	0.5612 (2)	0.54354 (11)	0.0224 (5)	
H2	0.4464	0.5574	0.5882	0.027*	
C3	0.39418 (11)	0.6732 (2)	0.51009 (12)	0.0263 (5)	
H3	0.3887	0.7479	0.5322	0.032*	
C4	0.36741 (11)	0.6791 (2)	0.44394 (12)	0.0260 (5)	
H4	0.3444	0.7579	0.4227	0.031*	
C5	0.37366 (11)	0.5743 (2)	0.40925 (11)	0.0232 (5)	
H5	0.3556	0.5785	0.3646	0.028*	
C6	0.40796 (10)	0.4615 (2)	0.44313 (11)	0.0199 (5)	
C7	0.45751 (11)	0.2659 (2)	0.47682 (11)	0.0202 (5)	
H7	0.4738	0.1779	0.4766	0.024*	
C8	0.40337 (11)	0.2955 (2)	0.35930 (11)	0.0206 (5)	
H8A	0.4220	0.2054	0.3595	0.025*	
H8B	0.4187	0.3611	0.3382	0.025*	
C9	0.33254 (11)	0.2861 (2)	0.32330 (11)	0.0209 (5)	
C10	0.29914 (11)	0.2108 (2)	0.34747 (11)	0.0208 (5)	
H10	0.3215	0.1614	0.3856	0.025*	
C11	0.23407 (11)	0.2072 (2)	0.31670 (11)	0.0222 (5)	
C12	0.20141 (12)	0.2768 (3)	0.25988 (12)	0.0285 (6)	
H12	0.1567	0.2742	0.2382	0.034*	
C13	0.23399 (13)	0.3496 (3)	0.23511 (12)	0.0338 (6)	
H13	0.2117	0.3959	0.1961	0.041*	
C14	0.29951 (12)	0.3554 (3)	0.26713 (11)	0.0291 (6)	
H14	0.3216	0.4073	0.2503	0.035*	
C15	0.19950 (12)	0.1274 (2)	0.34467 (12)	0.0257 (5)	
H15A	0.1702	0.0633	0.3126	0.031*	
H15B	0.2303	0.0730	0.3805	0.031*	
C16	0.10264 (11)	0.1968 (2)	0.35076 (11)	0.0223 (5)	
H16	0.0766	0.1318	0.3206	0.027*	
C17	0.13518 (11)	0.3561 (2)	0.42061 (11)	0.0221 (5)	
C18	0.14072 (12)	0.4591 (2)	0.46287 (11)	0.0251 (5)	
H18	0.1058	0.4870	0.4686	0.030*	
C19	0.19923 (12)	0.5187 (3)	0.49612 (12)	0.0285 (6)	
H19	0.2047	0.5892	0.5254	0.034*	
C20	0.25048 (12)	0.4777 (3)	0.48768 (12)	0.0300 (6)	
H20	0.2899	0.5214	0.5114	0.036*	
C21	0.24572 (12)	0.3754 (3)	0.44588 (12)	0.0283 (6)	
H21	0.2808	0.3474	0.4405	0.034*	
C22	0.18653 (11)	0.3158 (2)	0.41212 (11)	0.0227 (5)	
C23	0.31081 (17)	0.7967 (4)	0.64606 (15)	0.0501 (9)	
H23	0.2819	0.8643	0.6446	0.060*	
C24	0.4155 (2)	0.7252 (4)	0.6803 (2)	0.0749 (12)	
H24A	0.3929	0.6512	0.6510	0.112*	
H24B	0.4396	0.6883	0.7229	0.112*	
H24C	0.4443	0.7688	0.6672	0.112*	
C25	0.3954 (2)	0.9448 (4)	0.71704 (16)	0.0667 (12)	
H25A	0.3605	1.0025	0.7133	0.100*	
H25B	0.4211	0.9949	0.7020	0.100*	
H25C	0.4213	0.9192	0.7612	0.100*	

### Atomic displacement parameters (Å^2^)

**Table:**

	*U*^11^	*U*^22^	*U*^33^	*U*^12^	*U*^13^	*U*^23^
Zn1	0.01891 (15)	0.01906 (14)	0.01959 (14)	0.00064 (11)	0.00751 (11)	0.00179 (10)
Cl1	0.0220 (3)	0.0268 (3)	0.0201 (3)	−0.0046 (2)	0.0067 (2)	−0.0007 (2)
Cl2	0.0442 (4)	0.0243 (3)	0.0351 (3)	0.0130 (3)	0.0162 (3)	0.0053 (3)
O1	0.093 (2)	0.0474 (15)	0.0492 (14)	−0.0216 (15)	0.0061 (14)	−0.0035 (12)
N1	0.0187 (10)	0.0179 (10)	0.0211 (9)	0.0017 (8)	0.0088 (8)	0.0031 (8)
N2	0.0178 (10)	0.0180 (9)	0.0194 (9)	0.0005 (8)	0.0089 (8)	0.0005 (7)
N3	0.0229 (11)	0.0223 (10)	0.0239 (10)	−0.0048 (8)	0.0126 (9)	−0.0058 (8)
N4	0.0228 (10)	0.0218 (10)	0.0236 (10)	−0.0057 (8)	0.0116 (9)	−0.0045 (8)
N5	0.0455 (15)	0.0325 (13)	0.0262 (11)	−0.0073 (11)	0.0112 (11)	−0.0024 (10)
C1	0.0144 (11)	0.0178 (11)	0.0231 (11)	−0.0012 (8)	0.0085 (9)	0.0003 (9)
C2	0.0172 (12)	0.0240 (12)	0.0237 (12)	−0.0005 (9)	0.0078 (10)	−0.0013 (9)
C3	0.0247 (13)	0.0203 (12)	0.0344 (13)	0.0034 (10)	0.0143 (11)	−0.0034 (10)
C4	0.0224 (13)	0.0211 (12)	0.0328 (13)	0.0068 (10)	0.0116 (11)	0.0061 (10)
C5	0.0207 (12)	0.0224 (12)	0.0249 (12)	0.0035 (9)	0.0092 (10)	0.0037 (9)
C6	0.0160 (11)	0.0202 (12)	0.0238 (12)	−0.0021 (9)	0.0096 (9)	0.0001 (9)
C7	0.0168 (11)	0.0186 (11)	0.0248 (12)	0.0005 (9)	0.0094 (9)	0.0031 (9)
C8	0.0229 (12)	0.0219 (11)	0.0199 (11)	0.0008 (9)	0.0123 (10)	0.0003 (9)
C9	0.0247 (13)	0.0202 (11)	0.0203 (11)	−0.0013 (9)	0.0128 (10)	−0.0025 (9)
C10	0.0243 (12)	0.0180 (11)	0.0198 (11)	0.0005 (9)	0.0099 (10)	−0.0002 (9)
C11	0.0266 (13)	0.0183 (11)	0.0236 (12)	−0.0034 (9)	0.0132 (10)	−0.0067 (9)
C12	0.0236 (13)	0.0315 (14)	0.0244 (12)	−0.0035 (10)	0.0062 (10)	−0.0048 (10)
C13	0.0312 (15)	0.0420 (16)	0.0202 (12)	−0.0028 (12)	0.0051 (11)	0.0071 (11)
C14	0.0290 (14)	0.0343 (14)	0.0240 (12)	−0.0048 (11)	0.0124 (11)	0.0049 (11)
C15	0.0268 (13)	0.0235 (13)	0.0307 (13)	−0.0033 (10)	0.0166 (11)	−0.0057 (10)
C16	0.0218 (12)	0.0229 (12)	0.0227 (12)	−0.0063 (9)	0.0106 (10)	−0.0034 (9)
C17	0.0220 (12)	0.0212 (12)	0.0210 (11)	−0.0034 (9)	0.0082 (10)	0.0000 (9)
C18	0.0274 (13)	0.0227 (12)	0.0264 (12)	−0.0009 (10)	0.0135 (11)	−0.0037 (10)
C19	0.0316 (14)	0.0248 (13)	0.0251 (13)	−0.0028 (11)	0.0100 (11)	−0.0058 (10)
C20	0.0250 (13)	0.0295 (14)	0.0311 (14)	−0.0088 (11)	0.0093 (11)	−0.0077 (11)
C21	0.0235 (13)	0.0293 (14)	0.0323 (13)	−0.0060 (10)	0.0130 (11)	−0.0050 (11)
C22	0.0256 (13)	0.0206 (12)	0.0225 (12)	−0.0053 (10)	0.0117 (10)	−0.0033 (9)
C23	0.053 (2)	0.0425 (18)	0.0376 (17)	−0.0103 (16)	0.0071 (15)	0.0080 (14)
C24	0.075 (3)	0.078 (3)	0.083 (3)	0.007 (2)	0.047 (3)	−0.009 (2)
C25	0.094 (3)	0.043 (2)	0.0422 (19)	−0.0191 (19)	0.014 (2)	−0.0115 (15)

### Geometric parameters (Å, °)

**Table:**

Zn1—N1	2.022 (2)	C8—H8B	0.9900
Zn1—N4^i^	2.025 (2)	C9—C14	1.383 (3)
Zn1—Cl1	2.2544 (6)	C9—C10	1.399 (3)
Zn1—Cl2	2.2262 (7)	C10—C11	1.384 (3)
O1—C23	1.211 (4)	C10—H10	0.9500
N1—C7	1.321 (3)	C11—C12	1.394 (4)
N1—C1	1.394 (3)	C11—C15	1.506 (3)
N2—C7	1.349 (3)	C12—C13	1.381 (4)
N2—C6	1.391 (3)	C12—H12	0.9500
N2—C8	1.465 (3)	C13—C14	1.394 (4)
N3—C16	1.339 (3)	C13—H13	0.9500
N3—C22	1.389 (3)	C14—H14	0.9500
N3—C15	1.474 (3)	C15—H15A	0.9900
N4—C16	1.314 (3)	C15—H15B	0.9900
N4—C17	1.403 (3)	C16—H16	0.9500
N4—Zn1^i^	2.025 (2)	C17—C18	1.393 (3)
N5—C23	1.312 (4)	C17—C22	1.397 (3)
N5—C25	1.431 (4)	C18—C19	1.382 (3)
N5—C24	1.447 (5)	C18—H18	0.9500
C1—C6	1.395 (3)	C19—C20	1.396 (4)
C1—C2	1.397 (3)	C19—H19	0.9500
C2—C3	1.382 (3)	C20—C21	1.386 (4)
C2—H2	0.9500	C20—H20	0.9500
C3—C4	1.408 (3)	C21—C22	1.396 (3)
C3—H3	0.9500	C21—H21	0.9500
C4—C5	1.375 (3)	C23—H23	0.9500
C4—H4	0.9500	C24—H24A	0.9800
C5—C6	1.394 (3)	C24—H24B	0.9800
C5—H5	0.9500	C24—H24C	0.9800
C7—H7	0.9500	C25—H25A	0.9800
C8—C9	1.510 (3)	C25—H25B	0.9800
C8—H8A	0.9900	C25—H25C	0.9800
			
N1—Zn1—N4^i^	99.30 (8)	C10—C11—C12	119.4 (2)
N1—Zn1—Cl1	112.62 (6)	C10—C11—C15	119.7 (2)
N1—Zn1—Cl2	105.97 (6)	C12—C11—C15	120.9 (2)
N4^i^—Zn1—Cl1	101.28 (6)	C13—C12—C11	120.0 (2)
N4^i^—Zn1—Cl2	114.92 (6)	C13—C12—H12	120.0
Cl1—Zn1—Cl2	120.81 (3)	C11—C12—H12	120.0
C7—N1—C1	105.68 (18)	C12—C13—C14	120.2 (2)
C7—N1—Zn1	126.28 (15)	C12—C13—H13	119.9
C1—N1—Zn1	126.89 (15)	C14—C13—H13	119.9
C7—N2—C6	107.21 (19)	C9—C14—C13	120.4 (2)
C7—N2—C8	126.25 (19)	C9—C14—H14	119.8
C6—N2—C8	126.44 (19)	C13—C14—H14	119.8
C16—N3—C22	107.3 (2)	N3—C15—C11	113.3 (2)
C16—N3—C15	124.6 (2)	N3—C15—H15A	108.9
C22—N3—C15	127.7 (2)	C11—C15—H15A	108.9
C16—N4—C17	105.0 (2)	N3—C15—H15B	108.9
C16—N4—Zn1^i^	124.06 (16)	C11—C15—H15B	108.9
C17—N4—Zn1^i^	128.75 (16)	H15A—C15—H15B	107.7
C23—N5—C25	124.9 (3)	N4—C16—N3	113.5 (2)
C23—N5—C24	117.5 (3)	N4—C16—H16	123.3
C25—N5—C24	117.6 (3)	N3—C16—H16	123.3
N1—C1—C6	109.0 (2)	C18—C17—C22	121.3 (2)
N1—C1—C2	130.1 (2)	C18—C17—N4	129.7 (2)
C6—C1—C2	120.9 (2)	C22—C17—N4	109.0 (2)
C3—C2—C1	116.9 (2)	C19—C18—C17	117.1 (2)
C3—C2—H2	121.6	C19—C18—H18	121.5
C1—C2—H2	121.6	C17—C18—H18	121.5
C2—C3—C4	121.6 (2)	C18—C19—C20	121.5 (2)
C2—C3—H3	119.2	C18—C19—H19	119.3
C4—C3—H3	119.2	C20—C19—H19	119.3
C5—C4—C3	121.9 (2)	C21—C20—C19	122.1 (2)
C5—C4—H4	119.0	C21—C20—H20	118.9
C3—C4—H4	119.0	C19—C20—H20	118.9
C4—C5—C6	116.3 (2)	C20—C21—C22	116.3 (2)
C4—C5—H5	121.8	C20—C21—H21	121.9
C6—C5—H5	121.8	C22—C21—H21	121.9
N2—C6—C5	132.1 (2)	N3—C22—C21	133.0 (2)
N2—C6—C1	105.54 (19)	N3—C22—C17	105.2 (2)
C5—C6—C1	122.4 (2)	C21—C22—C17	121.8 (2)
N1—C7—N2	112.6 (2)	O1—C23—N5	126.3 (4)
N1—C7—H7	123.7	O1—C23—H23	116.8
N2—C7—H7	123.7	N5—C23—H23	116.8
N2—C8—C9	110.68 (19)	N5—C24—H24A	109.5
N2—C8—H8A	109.5	N5—C24—H24B	109.5
C9—C8—H8A	109.5	H24A—C24—H24B	109.5
N2—C8—H8B	109.5	N5—C24—H24C	109.5
C9—C8—H8B	109.5	H24A—C24—H24C	109.5
H8A—C8—H8B	108.1	H24B—C24—H24C	109.5
C14—C9—C10	118.9 (2)	N5—C25—H25A	109.5
C14—C9—C8	120.7 (2)	N5—C25—H25B	109.5
C10—C9—C8	120.4 (2)	H25A—C25—H25B	109.5
C11—C10—C9	121.0 (2)	N5—C25—H25C	109.5
C11—C10—H10	119.5	H25A—C25—H25C	109.5
C9—C10—H10	119.5	H25B—C25—H25C	109.5
			
N4^i^—Zn1—N1—C7	−101.91 (19)	C9—C10—C11—C15	−178.8 (2)
Cl2—Zn1—N1—C7	17.5 (2)	C10—C11—C12—C13	−0.6 (4)
Cl1—Zn1—N1—C7	151.65 (17)	C15—C11—C12—C13	179.9 (2)
N4^i^—Zn1—N1—C1	64.00 (19)	C11—C12—C13—C14	−0.9 (4)
Cl2—Zn1—N1—C1	−176.59 (17)	C10—C9—C14—C13	−0.2 (4)
Cl1—Zn1—N1—C1	−42.44 (19)	C8—C9—C14—C13	−178.1 (2)
C7—N1—C1—C6	−0.1 (2)	C12—C13—C14—C9	1.3 (4)
Zn1—N1—C1—C6	−168.31 (15)	C16—N3—C15—C11	128.1 (2)
C7—N1—C1—C2	−179.8 (2)	C22—N3—C15—C11	−59.4 (3)
Zn1—N1—C1—C2	12.0 (3)	C10—C11—C15—N3	112.2 (2)
N1—C1—C2—C3	−179.9 (2)	C12—C11—C15—N3	−68.3 (3)
C6—C1—C2—C3	0.5 (3)	C17—N4—C16—N3	−0.5 (3)
C1—C2—C3—C4	−0.3 (4)	Zn1^i^—N4—C16—N3	−164.84 (16)
C2—C3—C4—C5	0.0 (4)	C22—N3—C16—N4	0.6 (3)
C3—C4—C5—C6	0.0 (4)	C15—N3—C16—N4	174.3 (2)
C7—N2—C6—C5	−179.8 (2)	C16—N4—C17—C18	179.7 (2)
C8—N2—C6—C5	−3.4 (4)	Zn1^i^—N4—C17—C18	−17.1 (4)
C7—N2—C6—C1	0.0 (2)	C16—N4—C17—C22	0.3 (3)
C8—N2—C6—C1	176.4 (2)	Zn1^i^—N4—C17—C22	163.61 (17)
C4—C5—C6—N2	180.0 (2)	C22—C17—C18—C19	−0.3 (4)
C4—C5—C6—C1	0.1 (3)	N4—C17—C18—C19	−179.6 (2)
N1—C1—C6—N2	0.0 (2)	C17—C18—C19—C20	0.0 (4)
C2—C1—C6—N2	179.7 (2)	C18—C19—C20—C21	−0.1 (4)
N1—C1—C6—C5	179.9 (2)	C19—C20—C21—C22	0.4 (4)
C2—C1—C6—C5	−0.4 (4)	C16—N3—C22—C21	179.6 (3)
C1—N1—C7—N2	0.1 (3)	C15—N3—C22—C21	6.1 (4)
Zn1—N1—C7—N2	168.44 (15)	C16—N3—C22—C17	−0.3 (3)
C6—N2—C7—N1	−0.1 (3)	C15—N3—C22—C17	−173.8 (2)
C8—N2—C7—N1	−176.5 (2)	C20—C21—C22—N3	179.4 (3)
C7—N2—C8—C9	115.4 (2)	C20—C21—C22—C17	−0.7 (4)
C6—N2—C8—C9	−60.3 (3)	C18—C17—C22—N3	−179.4 (2)
N2—C8—C9—C14	125.4 (2)	N4—C17—C22—N3	0.0 (3)
N2—C8—C9—C10	−52.4 (3)	C18—C17—C22—C21	0.7 (4)
C14—C9—C10—C11	−1.3 (4)	N4—C17—C22—C21	−179.9 (2)
C8—C9—C10—C11	176.5 (2)	C25—N5—C23—O1	−176.3 (3)
C9—C10—C11—C12	1.7 (3)	C24—N5—C23—O1	2.7 (5)

Symmetry codes: (i) −*x*+1/2, −*y*+1/2, −*z*+1.
